# PSMC2 Regulates Cell Cycle Progression Through the p21/Cyclin D1 Pathway and Predicts a Poor Prognosis in Human Hepatocellular Carcinoma

**DOI:** 10.3389/fonc.2021.607021

**Published:** 2021-02-26

**Authors:** Yiwei Liu, Hairong Chen, Xiangcheng Li, Feng Zhang, Lianbao Kong, Xuehao Wang, Jin Bai, Xiaofeng Wu

**Affiliations:** ^1^Hepatobiliary Center, The First Affiliated Hospital of Nanjing Medical University, Nanjing, China; ^2^Key Laboratory of Liver Transplantation, Chinese Academy of Medical Sciences, Nanjing, China; ^3^NHC Key Laboratory of Living Donor Liver Transplantation (Nanjing Medical University), Nanjing, China; ^4^Department of Occupational Medicine and Environmental Health, School of Public Health, Nanjing Medical University, Nanjing, China; ^5^Cancer Institute, Xuzhou Medical University, Xuzhou, China; ^6^Center of Clinical Oncology, Affiliated Hospital of Xuzhou Medical University, Xuzhou, China

**Keywords:** PSMC2, hepatocellular carcinoma, p21, cell proliferation, carcinogenesis

## Abstract

Proteasome 26S subunit ATPase 2 (PSMC2) plays a pathogenic role in various cancers. However, its function and molecular mechanism in hepatocellular carcinoma (HCC) remain unknown. In this study, tissue microarray (TMA) analysis showed that PSMC2 is highly expressed in HCC tumors and correlates with poor overall and disease-free survival in HCC patients. Multivariate Cox regression analysis revealed that PSMC2 is an independent prognostic factor for HCC patients. Furthermore, our results showed that PSMC2 knockdown inhibited cell proliferation and suppressed tumorigenesis *in vivo*. Knockdown of PSMC2 increased the expression of p21 and therefore decreased the expression of cyclin D1. Dual-luciferase reporter assays indicated that depletion of PSMC2 significantly enhanced the promoter activity of p21. Importantly, PSMC2 knockdown-induced phenotypes were also rescued by downregulation of P21. Taken together, our data suggest that PSMC2 promotes HCC cell proliferation and cell cycle progression through the p21/cyclin D1 signaling pathway and could be a promising diagnostic and therapeutic target for HCC patients.

## Introduction

Hepatocellular carcinoma (HCC) is one of most malignant cancers and has a poor prognosis, ranking fifth in terms of global incidence and second in terms of mortality rate among males (1) The majority of HCC cases occurs in patients with advanced fibrosis, mainly due to hepatitis B or C virus (HBV or HCV) infection or alcohol abuse (2) Patients with HCC commonly have a poor prognosis and a high recurrence rate. This is mainly because the majority of HCC patients is diagnosed at advanced stages (3–7). Although risk factors for recurrence, including tumor size, alpha fetoprotein (AFP), tumor differentiation, cirrhosis, surgical margin, serum HBV viral load, and metabolic syndrome, have been proposed to be associated with the prognosis of HCC, their clinical application is limited (7). Recurrence after resection or transplantation is unpredictable. Therefore, the investigation of HCC-related genes and their molecular biological mechanisms has important clinical value in identifying patients with a poor prognosis and exploring effective treatment strategies for HCC.

Proteasome 26S subunit ATPase 2 (PSMC2) is the highest ranked candidate among the Copy-number alterations Yielding Cancer Liabilities Owing to Partial loss (CYCLOPS) genes, which are a special subset of essential genes involved in cancer cell viability (8). The 26S proteasome complex is a well-known polyproteinase consisting of a 20S core particle and a 19S regulatory particle (RP) that can rapidly disrupt key intracellular regulatory proteins (e.g., transcription factors and cell cycle regulators) (9–11). PSMC2, located at 7q22.1-q22.3 in the genome, is a key member of the 19S RP of the 26S proteasome complex (12–14). Of note, PSMC2 has also been identified as a vital factor in tumor development and progression. For instance, it has been reported that PSMC2 is implicated in the progression of ovarian cancer (8), pancreatic cancer (15), colorectal cancer (16), and osteosarcoma (16–18). However, research on PSMC2 is limited to functional experiments, and no studies have investigated the underlying mechanisms of the function of PSMC2 in cancer. Therefore, in this study, we described the important role of PSMC2 in the progression of HCC, which has never been reported.

In the current study, we investigated the expression of PSMC2 in a tissue microarray (TMA) and explored its potential role and molecular mechanism in regulating HCC malignant features. Our research showed that PSMC2 was markedly upregulated in patient HCC tissues compared with adjacent non-cancerous tissues, and its upregulation was positively correlated with worse survival outcomes in HCC patients. In addition, *in vivo* and *in vitro* experiments showed that PSMC2 promotes HCC cell cycle progression through the p21/cyclin D1 signaling pathway.

## Materials and Methods

### Patient Specimens and Construction of Tissue Microarrays

TMA consists of 220 HCC samples and corresponding adjacent non-cancerous tissues, collected by the Hepatobiliary Center of the First Affiliated Hospital of Nanjing Medical University (Nanjing, Jiangsu Province, China). All patients underwent radical surgery at Nanjing Medical University from January 2009 to December 2010. Patients' clinical data were obtained from the hospital's medical records, including sex, age, tumor size, preoperative AFP, tumor-node-metastasis (TNM) stage, date of surgery, place of birth, and marriage. The patient studies were conducted in accordance with the Declaration of Helsinki. The use of these specimens and data for research purposes was approved by the ethics committee of the hospital, and informed consent was obtained from all patients.

Of the 220 HCC patients who were retrospectively analyzed, 149 were males and 71 were females with an average age of 54.8 years (24–79 years). A complete postoperative follow-up record was obtained for each patient. Survival time was calculated from the date of surgery to the date of death or last follow-up. In addition, the date of death was obtained from postoperative follow-up records and verified by the local civil affairs department.

Overall survival (OS) refers to the time from the start of surgery to the death of the patient or the time of last follow-up. Disease-free survival (DFS) refers to the occurrence of recurrent tumors, death, or last follow-up from the time of surgery. Our last follow-up was October 2018.

### Immunohistochemistry (IHC)

IHC was implemented following a standard streptavidin-peroxidase method as previously reported (19). Anti-PSMC2 (1:1,000 dilution; HPA019238, Sigma, USA) was used for IHC according to the avidin-biotin complex method. After microwave antigen retrieval, tissues were incubated with primary antibody at 4°C overnight. Following incubation with secondary antibody, the sections were developed in a diaminobenzidine solution under a microscope and counterstained with hematoxylin. Negative control slides were included in all assays.

### Cell Lines and Cell Culture

The MHCC97-L (established at the Liver Cancer Institute, Zhongshan Hospital, Fudan University) (20) and SMMC-7721 cell lines (purchased from the Shanghai Institute of Biochemistry and Cell Biology, Chinese Academy of Science, Shanghai, China) were used in this study. They were cultured in DMEM supplemented with 10% fetal bovine serum, 100 U/ml of penicillin, and 100 μg/ml of streptomycin and cultured in a 37°C humidified incubator with 5% CO_2_.

### Small Interfering RNA (siRNA) and Transient Transfections

SiRNA specific for PSMC2 (siPSMC2) and non-specific control (siCtrl) were purchased from GenePharma (Shanghai, China) and transfected with siLentFect Lipid Reagent (Bio-Rad Laboratories, Inc.) in accordance with the manufacturer's protocol. The cells were grown to 30–40% confluency. Six hours after transfection, the medium containing transfection reagents was replaced with fresh medium. The siRNA sequences were described as follows (8):

SiPSMC2#1 sense: 5′-GCUGUAAAUAAGGUCAUUAUU;
SiPSMC2#2 sense: 5′-GCCAGGUGUACAAAGAUAAUU;
SiPSMC2#3sense: 5′-GGACCCACAUAUUUAAGAUUU;
Sip21 sense: 5′-CCUCUGCAUUAGAAUUAUTT;
SiCtrl sense: 5′-GGCUACGUCCAGGAGCGCA.

### Stable Cell Line Establishment

A PSMC2 knockdown lentivirus was purchased from Shanghai GeneChem Company. The shRNA target sequences were described as follows: sh PSMC2 sense: CAGGGAGATTGGATAGAAA; shCtrl sense: TTCTCCGAACGTGTCACGT. MHCC97-L cells were infected with lentivirus for 48 h and then selected with 2 ng/ml of puromycin for 2 weeks.

### Western Blot Analysis

Western blot analysis was conducted in accordance with a previous study (21). The specific primary antibodies against PSMC2 (HPA019238) were purchased from Sigma. Antibodies against GAPDH (60004-1-Ig) and p21 (10355-1-AP) were purchased from Proteintech. Antibodies against cyclin D1 (#2978), cleaved PARP (#5625), cleaved caspase 3 (#9661P), cleaved caspase 7 (#9491P), and BCL-XL (#2764) were obtained from Cell Signaling Technology.

### RNA Extraction and Quantitative Real-Time PCR (qRT-PCR)

Total RNA was extracted from SMMC-7721 and MHCC97-L cells using TRIzol reagent (Invitrogen, California, USA). cDNA was generated using HiScript Q RT SuperMix for qPCR (+gDNA wiper) (Vazyme Biotech, Nanjing, China). Real-time PCR was conducted on a LightCycler® 480 using SYBR Green Real-Time PCR Master Mix (Vazyme Biotech, Nanjing, China). All primers used in this study are listed as follows:

PSMC2: 5′-CAGAGGCTGGTATGTTTG-3′ (forward) and 5′-CCTTCAGGGTTCAGTTGT-3′ (reverse);
p21: 5′-TTTCTCTCGGCTCCCCATGT-3′ (forward) and 5′-GCTGTATATTCAGCATTGTGGG-3′ (reverse);
GAPDH: 5′-TGACTTCAACAGCGACACCCA-3′ (forward) and 5′-CACCCTGTTGCTGTAGCCAAA-3′ (reverse).

### Cell Proliferation Assay

Cell Counting Kit-8 (CCK-8) assays were conducted in accordance with the manufacturer's protocol to determine the regulation of cell proliferation by PSMC2. SMMC-7721 and MHCC97-L cells were transiently transfected with siRNAs targeting PSMC2 (siPSMC2#1, siPSMC2#2) or control siRNA (siCtrl). Two days after transfection, ~5 × 103 cells were seeded in each well of 96-well plates, and CCK-8 solution was added 1, 2, 3, and 4 days after. Cells were incubated at 37°C for 2 h after 10 μl of CCK-8 solution was added. The absorbance at 450 nm was measured.

Celigo Imaging Cytometer (Nexcelom Bioscience, Lawrence, MA) was used to count the number of surviving cells. Seventy-two hours after infection, the cells were collected and seeded into 96-well plates at a density of 1,000 cells per well. Cell growth curves and fluorescent photomicrographs were taken by measuring cells with green fluorescence with a cytometer in the following 5 days.

For colony formation assay, 800 MHCC97-L cells were seeded in a six well-plate for 14 days, washed twice with PBS, fixed with 4% paraformaldehyde for 30 min, and stained with Giemsa for 20 min. The number of colonies was counted visually.

### Apoptosis

The cells were collected after transfection with siRNA for 48 h. Then, they were washed twice with PBS and resuspended in binding buffer. Apoptosis assay was conducted using the Annexin V-FITC/PI apoptosis detection kit (Nanjing KeyGen Biotech, Inc.) in accordance with the manufacturer's protocol. Sequentially, the cells were stained with Annexin V-FITC and PI at room temperature for 15 min and then analyzed by flow cytometry (BD, FACSCanto™ II).

### Cell Cycle Analysis

Forty-eight hours after transfection, the cells were starved for 24 h to synchronize the cells and induced to re-enter the cell cycle by incubating in a medium containing 10% fetal bovine serum for 6 h. The cells were then collected, washed twice with PBS, and fixed overnight in pre-cooled 70% ethanol at 4°C. On the next day, the cells were washed twice with PBS and incubated with RNase A in a 37°C water bath for 30 min. Then, PI was added to the cells at 4°C for 30 min in the dark. Finally, all samples were analyzed by flow cytometry (BD, FACSCanto™ II).

### Bioinformatic Analysis

To determine the correlation of PSMC2 mRNA expression with p21 target genes, we analyzed the datasets from published studies. Data were deposited at the Gene Expression Omnibus (GEO, GSE36376) database (22). GSE36376 contains data for 240 HCC tissues and 193 adjacent non-cancerous tissues. The average of the probes for the analyzed genes was used in all analyses.

### Dual-Luciferase Reporter Assays

SiPSMC2, siCtrl, p21 promoter plasmid, and renilla luciferase (Rluc) plasmid were transfected into MHCC97-L cells by using the siLentFect Lipid Reagent (Bio-Rad Laboratories, Inc.) and Lipofectamine 2,000 transfection reagent (Life Technologies, Shanghai, China). At 48 h post-transfection, the cells were lysed and analyzed for firefly luciferase and Rluc activity using the Dual-Luciferase Reporter Assay System (Promega). Rluc activity was used for normalization. The plasmid of p21-luc (−2,400/+11) was a gift from Dr. Baiqu Huang (The Institute of Genetics and Cytology, Northeast Normal University).

### Experimental Animal Model and Subject Details

Male BALB/c nude mice (6–8 weeks old) were purchased from Nanjing Gempharmatech Technology Co., Ltd. (Nanjing, China). All animal experiments were approved by the animal care and use committee. The tumor volume was calculated using the formula V = a × (b × b)/2, where a is the largest and b is the smallest diameter. Twenty-five days later, the mice were killed, and the tumors were weighted and processed to detect the expression of PSMC2, p21, and Ki67 by IHC analysis.

### Statistical Analysis

All statistical analyses were performed using GraphPad Prism 7 (Chicago, CA) and SPSS 25 statistical software package (SPSS Inc., Chicago, IL). The paired Wilcoxon test was used to assess the significance of PSMC2 staining in HCC tissues and corresponding adjacent non-cancerous tissues. OS and DFS were calculated by the Kaplan-Meier method and analyzed by the log-rank test. The χ2 test was performed to evaluate the relationship between PSMC2 expression and clinicopathological parameters. Univariate and multivariate analyses were based on the Cox proportional hazards regression model. The unpaired *t*-test was used to determine the statistical significance of differences between groups. “*” indicates a *P* < 0.05, which was considered statistically significant.

## Results

### PSMC2 Is Upregulated in HCC Tissues and Is an Independent Prognostic Factor for HCC

**Figure 1:**
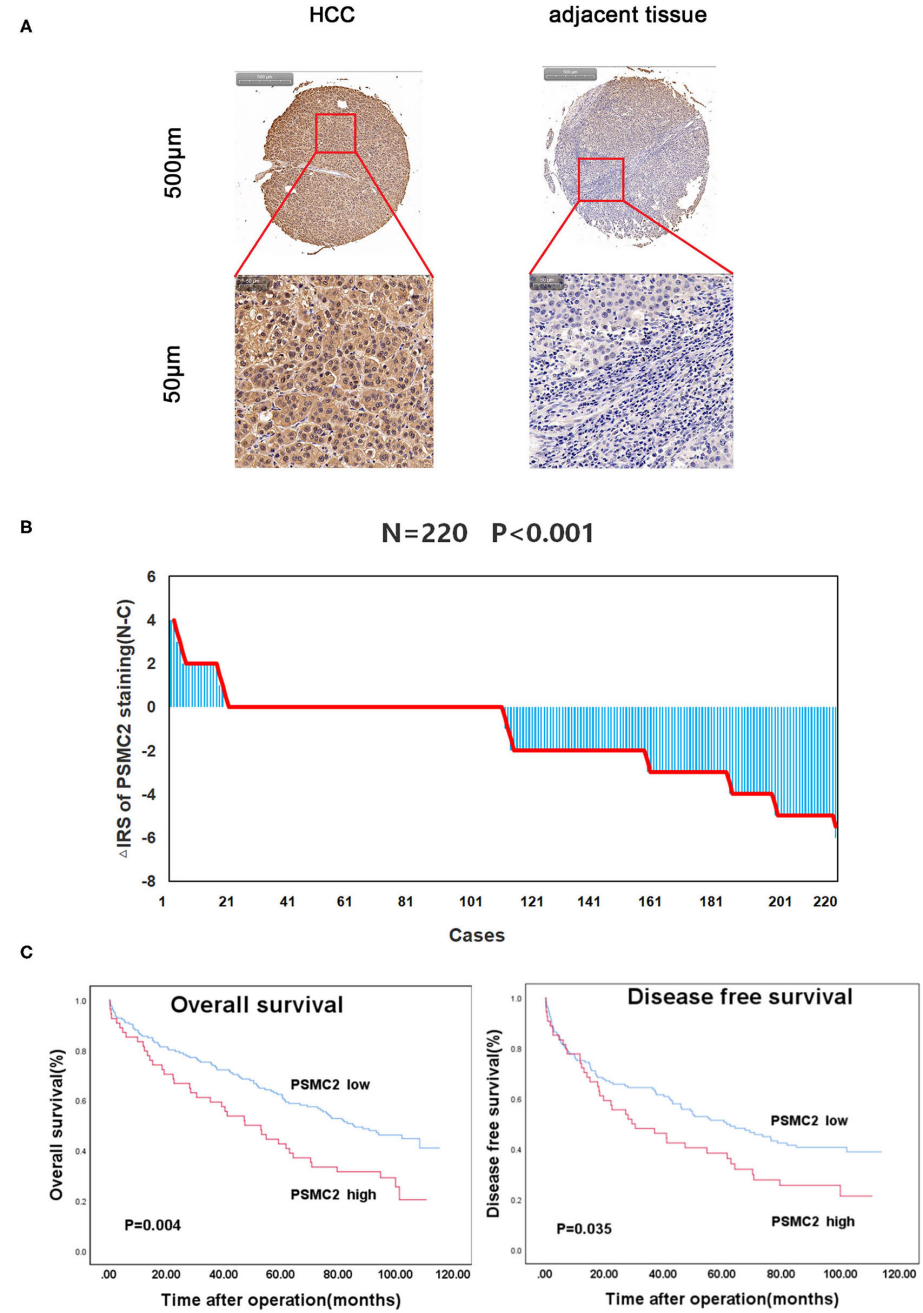
PSMC2 is highly expressed in HCC tumors and correlated with overall survival and disease-free survival for HCC patients. **(A)** PSMC2 immunostaining in TMAs are shown. Note: top panel, scale 500 μm; bottom panel, scale 50 μm. **(B)** The distribution of the difference in staining intensities of PSMC2 in HCC tissues compared with corresponding adjacent noncancerous tissue. N, paired adjacent noncancerous tissues. C, HCC tissues. PSMC2 expression levels were significantly higher in HCC compared with corresponding adjacent noncancerous tissues (Wilcoxon test, *P* < 0.001). **(C)** High PSMC2 expression is associated with poorer overall survival (*P* = 0.004, log-rank test) and disease-free survival for HCC patients (*P* = 0.035, log-rank test).

To investigate the role of PSMC2 in the development of HCC, we applied IHC to evaluate endogenous PSMC2 expression in the TMAs (containing 220 HCC samples and corresponding adjacent non-cancerous tissues). PSMC2 was located in the nucleus and cytoplasm (Figure 1A). A paired Wilcoxon test of paired tumor tissue and adjacent non-cancerous tissue from 220 cases revealed that PSMC2 protein expression was dramatically upregulated in cancerous tissue compared with adjacent non-cancerous tissue (*P* < 0.001, Figure 1B). Next, we analyzed the correlations between PSMC2 expression and characteristics of HCC and found that high PSMC2 protein expression was significantly correlated with tumor diameter (*P* = 0.002) and sex (*P* < 0.013, Table 1). In particular, we used Kaplan-Meier survival analysis and the log-rank test to examine whether PSMC2 expression was correlated with overall survival (OS) and disease-free survival (DFS) for HCC patients (Figure 1C). The results revealed that high PSMC2 levels were correlated with poor OS (*P* = 0.004) and DFS (*P* = 0.035).

**Table 1:** PSMC2 staining and clinicopathological characteristics of 220 HCC patients.

**Variables**	**PSMC2 staining**			
	**Low (%)**	**High (%)**	**Total**	P*
**Age**				
≤55	87 (78.3)	24 (21.6)	111	0.309
>55	79 (72.4)	30 (27.5)	109	
**Sex**				
Male	105 (70.5)	44 (29.5)	149	**0.013**				
Female	61 (85.9)	10 (14.1)	71	
**Preoperative AFP (ng/mL)**				
≤20	60 (72.3)	23 (27.7)	83	0.333
>20	90 (78.3)	25 (21.7)	115	
**ALT(U/L)**				
≤40	92 (75.5)	30 (24.5)	122	0.983
>40	67 (75.3)	22 (24.7)	89	
**Liver cirrhosis**				
Yes	111 (73.0)	41 (27.0)	152	0.211
No	55 (80.8)	13 (19.2)	68	
**Tumor size (cm)**				
≤5	112 (82.4)	24 (17.6)	136	**0.002**				
>5	54 (64.3)	30 (35.7)	84	
**Tumor number**				
Single	146 (75.2)	48 (24.8)	194	0.978
Multiple	18 (75.0)	6 (25.0)	24	
**TNM**				
I	52 (74.2)	18 (25.8)	70	0.781
II/III	111 (76.1)	35 (23.9)	146	
**Differentiation**				
I/II	98 (78.4)	27 (21.6)	125	0.388
III/IV	63 (73.3)	23 (26.7)	86	
**Tumor recurrence**				
Yes	92 (74.8)	31 (25.2)	123	0.428
No	59 (79.8)	15 (20.2)	74	

* *P values are from χ^2^ test. The bold values are considered as statistically significant*.

*Some cases were not available for the information*.

Finally, to further examine whether PSMC2 expression was an independent prognostic factor for HCC, we used univariate and multivariate Cox regression models to confirm the prognostic value of PSMC2 expression in HCC. Univariate Cox regression analysis suggested that PSMC2 expression, tumor size, and tumor-node-metastasis (TNM) stage were significant prognostic factors for HCC patients' OS and DFS (Table 2). In the multivariate Cox regression model, our data further confirmed that PSMC2 expression remained an independent significant prognostic biomarker for OS (*P* = 0.011, hazard ratio (HR) = 1.687, 95% confidence interval (CI) = 1.130-2.521) and DFS (*P* = 0.034, HR = 1.541, 95% CI = 1.033–2.299) for HCC patients after adjusting for age, sex, tumor size, tumor differentiation, TNM stage, and liver cirrhosis (Table 3). Collectively, our results confirmed that PSMC2 expression may serve as a potential independent prognostic factor for OS and DFS in HCC patients.

**Table 2:** Univariate Cox proportional regression analysis on overall and disease-free survival of 220 HCC patients.

**Variables**	**Overall survival**			**Disease-free survival**		
	**Hazard ratio**	**95% CI^**†**^**	P*	**Hazard ratio**	**95% CI^**†**^**	P*
**PSMC2**						
Low	1.000			1.000		
High	1.717	1.181–2.499	**0.005**	1.490	1.025–2.167	**0.037**
**Age**						
≤55 years	1.000			1.000		
>55 years	1.075	0.760–1.521	0.682	0.942	0.666–1.332	0.733
**Sex**						
Male	1.000			1.000		
Female	1.350	0.921–1.979	0.124	1.281	0.874–1.878	0.205
**Tumor size**						
≤5 cm	1.000			1.000		
>5 cm	1.803	1.273–2.552	**0.001**	1.648	1.164–2.333	**0.005**
**Liver cirrhosis**						
Yes	1.000			1.000		
No	1.307	0.907–1.883	0.151	1.221	0.838–1.759	0.283
**Differentiation**						
I/II	1.000			1.000		
III/IV	0.862	0.599–1.240	0.424	0.895	0.622–1.288	0.550
**TNM**						
I	1.000			1.000		
II/III	1.501	1.015–2.218	**0.042**	1.527	1.033–2.256	**0.034**

† *CI, confidence interval. The bold values are considered as statistically significant*.

*Some cases were not available for the information*.

**Table 3:** Multivariate Cox regression analysis on overall and disease-free survival of 220 HCC patients.

**Variables**	**Overall survival**			**Disease-free survival**		
	**Hazard ratio**	**95% CI^**†**^**	***P***	**Hazard ratio**	**95% CI^**†**^**	***P***
PSMC2	1.687	1.130–2.521	**0.011**	1.541	1.033–2.299	**0.034**
Age	1.036	0.717–1.497	0.850	0.927	0.644–1.336	0.686
Sex	1.176	0.791–1.749	0.422	1.168	0.785–1.740	0.444
Tumor size	1.524	1.039–2.234	**0.031**	1.405	0.963–2.050	0.078
Differentiation	0.863	0.597–1.247	0.433	0.874	0.605–1.262	0.472
TNM	1.421	0.940–2.146	0.095	1.492	0.989–2.251	0.057
Liver cirrhosis	0.864	0.581–1.285	0.470	0.888	0.599–1.315	0.552

* *Coding of variables: PSMC2 was coded as 0 (low), and 1 (high). Age was coded as 0 (≤55 years), and 1 (>55years). Sex was coded as 0 (male), and 1 (female). Tumor size was coded as 0 (≤5 cm), and 1 (>5 cm). Tumor differentiation was coded as 0 (I and II), and 1 (III and IV). TNM was coded as 0 (I), and 1 (II and III). Liver cirrhosis was coded as 0 (NO), and 1 (YES). CI, confidence interval. The bold values are considered as statistically significant. Some cases were not available for the information*.

### Downregulation of PSMC2 Inhibits the Proliferation of HCC Cells

**Figure 2:**
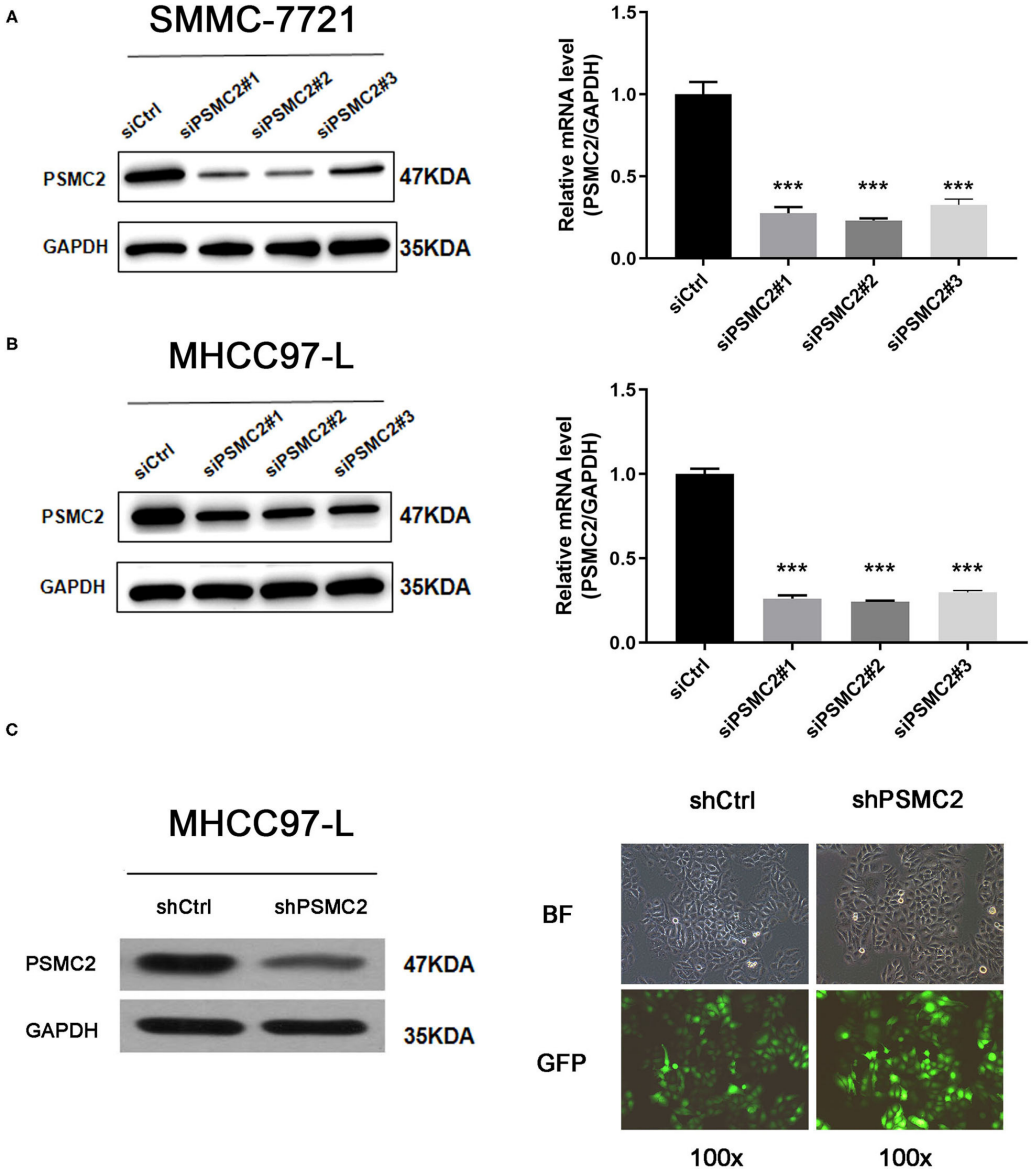
Transient transfection and construction of stable cell lines. **(A,B)** Knockdown of PSMC2 was confirmed at the protein level and mRNA level in SMMC-7721 and MHCC97-L cells by western blotting and real-time PCR. **(C)** Efficiency of PSMC2 silencing in MHCC97-L cells, as measured by Western blot. Infection efficiency of MHCC97-L cells with shRNA or shCtrl lentiviral vectors. Cells were assessed by fluorescent microscopy and light microscopy at day 3 post-infection. It is apparent that more than 80% of cells expressed GFP. Magnification, 100x. Representative images of the cultures are shown. ***P < 0.001.

Given that the TMA analysis showed that PSMC2 expression was associated with HCC progression, we further assessed the biological functions and molecular mechanisms of PSMC2 in HCC progression. SMMC-7721 and MHCC97-L cells were transiently transfected with siRNAs targeting PSMC2 (siPSMC2#1, siPSMC2#2, siPSMC2#3) or control siRNA (siCtrl). Then, Western blot analysis and qRT-PCR analysis were performed to determine the expression of PSMC2 in SMMC-7721 and MHCC97-L cells (Figures 2A,B). The results revealed that PSMC2 expression was significantly reduced in cells transfected with PSMC2 siRNA compared with those transfected with the control siRNA. We selected siPSMC2#1 and siPSMC2#2 for subsequent research. We also generated MHCC97-L-shCtrl and MHCC97-L-shPSMC2 cells (Figure 2C). Because the expression of PSMC2 was associated with tumor size, we assessed whether PSMC2 affected the apoptosis and proliferation of HCC cells.

**Figure 3:**
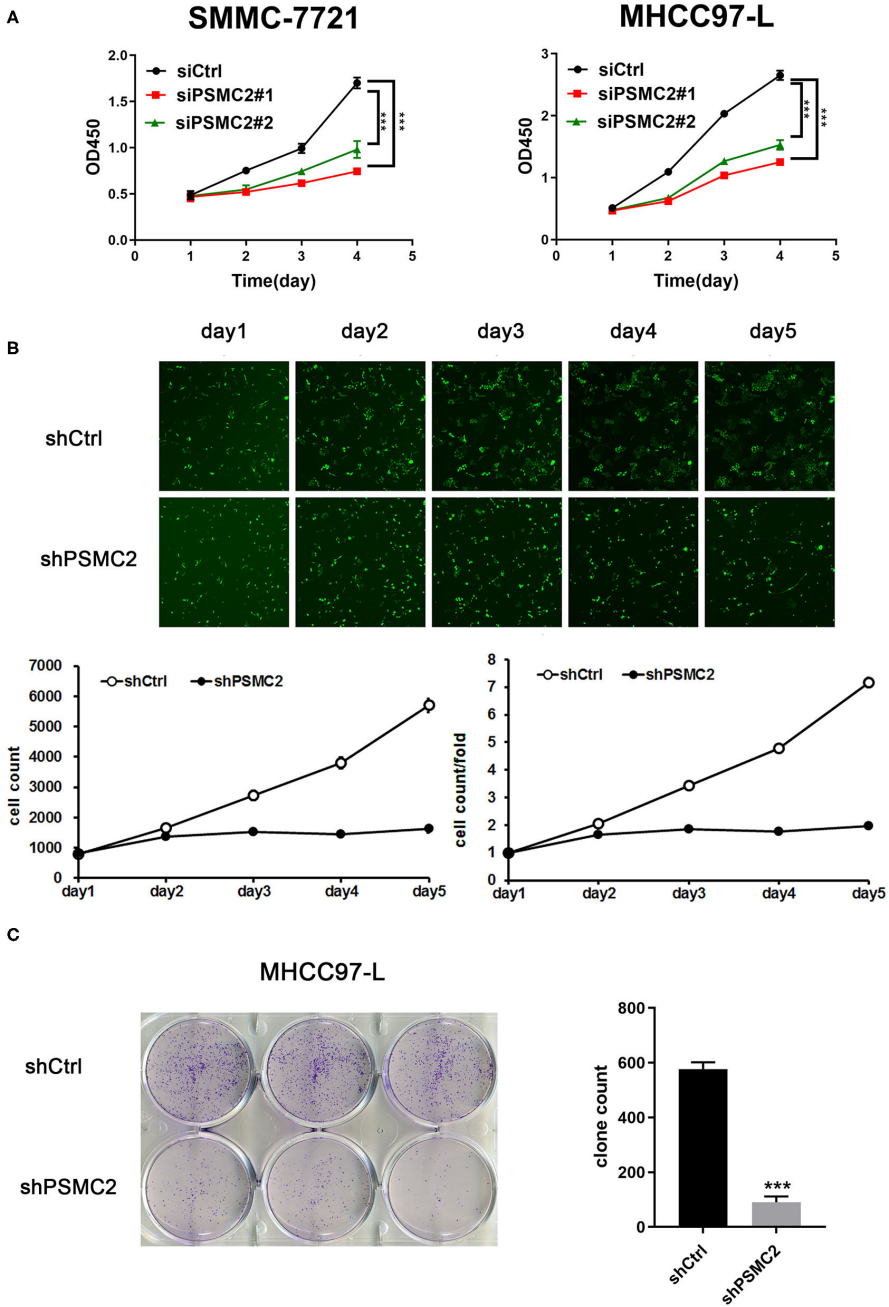
PSMC2 facilitates cell proliferation of HCC cells. **(A)** Knockdown of PSMC2 reduces the proliferation rate of SMMC-7721 and MHCC97-L cells. **(B)** Celigo cell counting was performed to measure the rate of cell proliferation. Fluorescent photomicrographs were taken for cells expressing green fluorescence protein at the indicated times. **(C)** PSMC2 knockdown inhibits the capacity of colony formation in MHCC97-L cells. Data are shown as mean ± standard deviations. ***P < 0.001.

With the Cell Counting Kit-8 (CCK-8) cell proliferation assay, we found that cell proliferation was decreased in cell lines transfected with PSMC2 siRNAs compared with those transfected with the control siRNA (Figure 3A). However, the CCK-8 cell proliferation assay results were influenced by the number of cells and the induction of apoptosis, reflecting the limited value of proliferation as a metric. Thus, PSMC2 knockdown MHCC97-L cells were subjected to Celigo cell counting and colony formation assays. The growth curve, quantified, and generated with a Nexcelom Celigo Image Cytometer, showed that the growth rate was significantly decreased following shRNA-mediated knockdown of PSMC2 in MHCC97-L cells (Figure 3B). In addition, colony formation assays showed that PSMC2 expression was positively correlated with cell growth (Figure 3C). In conclusion, these results indicate that PSMC2 is essential for the proliferation of HCC cells.

### Downregulation of PSMC2 Promotes Apoptosis of HCC Cells

**Figure 4:**
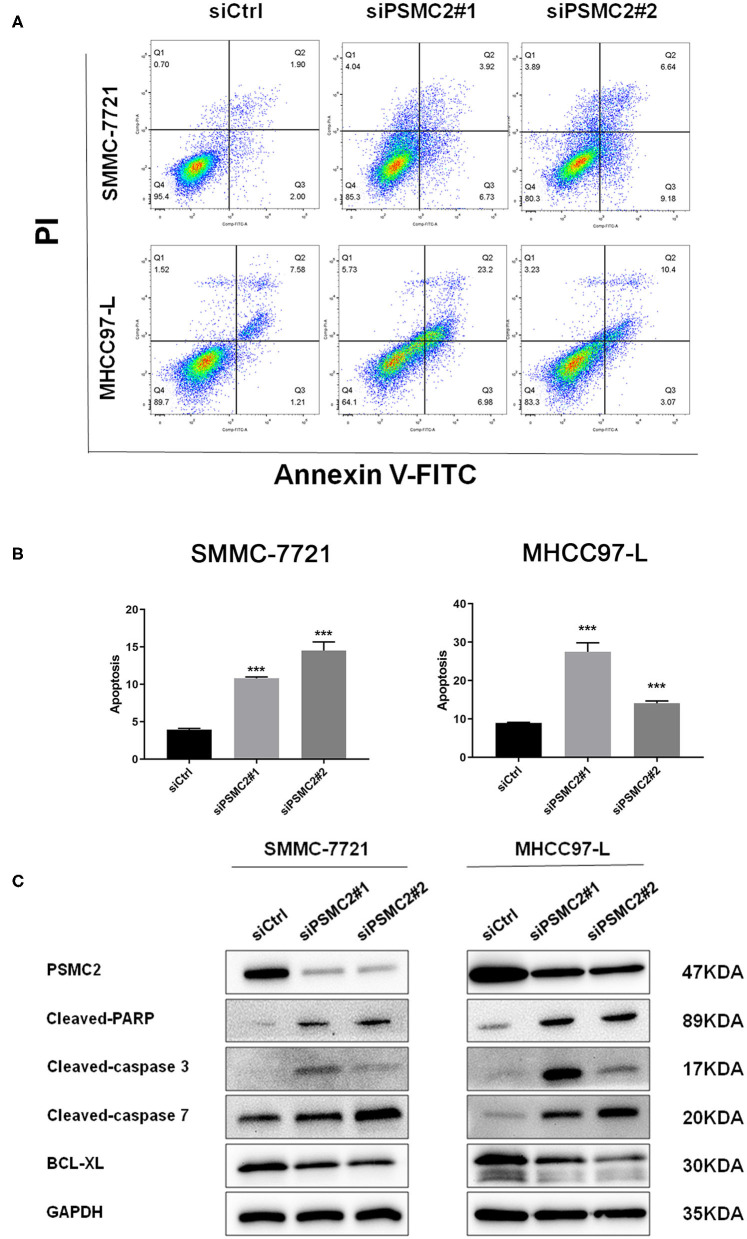
PSMC2 inhibits cell apoptosis of HCC cells. **(A)** Knockdown of PSMC2 increased HCC cell apoptosis, as detected by flow cytometric analysis following Annexin FITC and PI staining. **(B)** Statistics on the percentage of Apoptotic cells. **(C)** The expression of genes related with apoptosis was detected by western blotting in protein level. GAPDH was used as a reference control. All experiments were carried out three times independently. Data are mean ± standard deviation. ***P < 0.001.

Cell apoptosis also has a significant effect on cell proliferation, so we next examined the effect of PSMC2 on apoptosis using flow cytometry analysis. Morphological changes in SMMC-7721 and MHCC97-L cells were assessed by Annexin V-FITC/PI double-staining assays. We observed that PSMC2 knockdown increased the apoptosis rate compared with that in the corresponding control group (Figures 4A,B). In addition, Western blot analysis showed that PSMC2 knockdown dramatically increased the expression of apoptotic factors, including cleaved-PARP, cleaved caspase 3, and cleaved caspase 7, and decreased the expression of BCL-XL (Figure 4C).

### Downregulation of PSMC2 Inhibits the G1/S Phase Transition of the Cell Cycle

**Figure 5:**
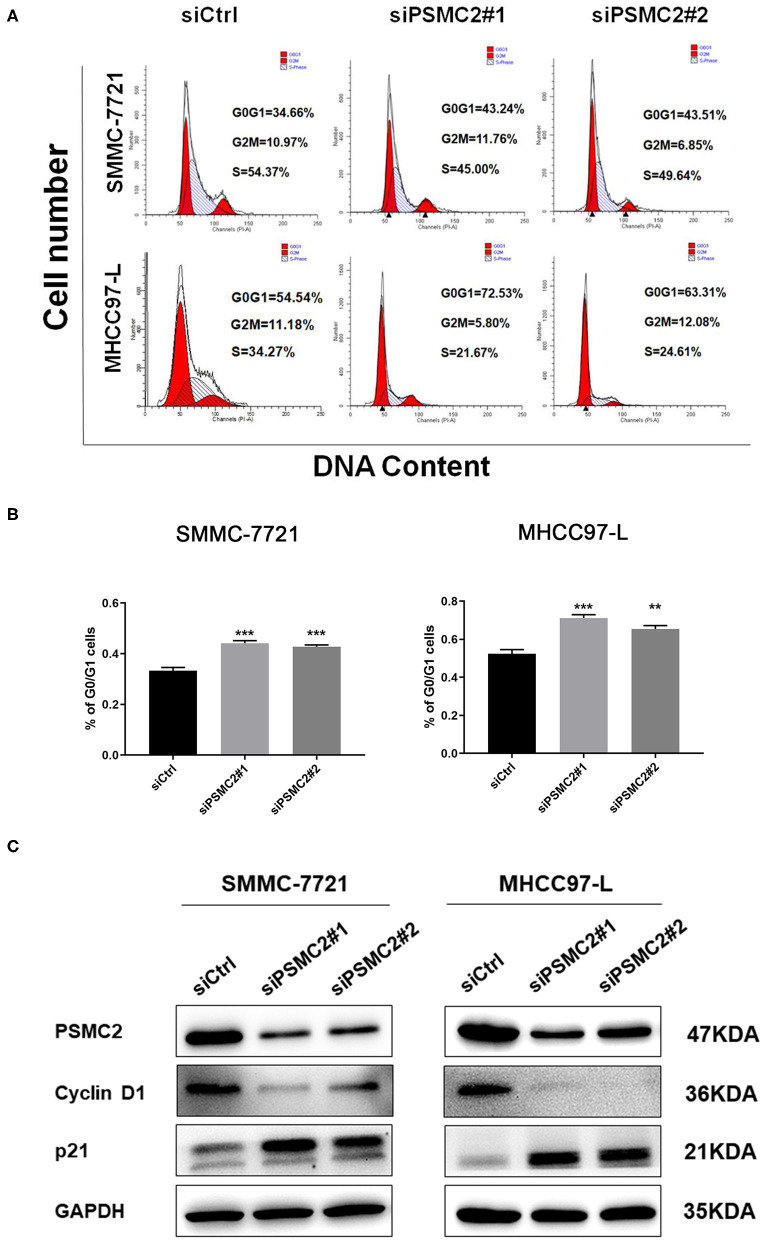
PSMC2 accelerates cell cycle progression in HCC cells. **(A)** Knockdown of PSMC2 increased G0/G1 phase cell population but decreased S phase cell population, as shown by flow cytometric analysis followed by Annexin PI staining. **(B)** Statistics on the percentage of G0/G1cells. **(C)** The expression of genes related with cell cycle was detected by western blotting in protein level. GAPDH was used as a reference control. All experiments were carried out three times independently. Data are mean ± standard deviation. **P < 0.01, ***P < 0.001.

To explore the possible mechanism by which PSMC2 knockdown inhibits HCC cell proliferation, we performed cell cycle analysis to examine whether PSMC2 knockdown induced the inhibition of HCC cell proliferation due to arrest at a certain stage. We synchronized cells for 24 h by serum starvation and collected cells 6 h after serum addition. Flow cytometry analysis showed that PSMC2 downregulation significantly increased the proportion of cells in the G0/G1 phase and decreased the proportion of cells in the S phase (Figures 5A,B). Therefore, PSMC2 downregulation appears to inhibit the G1/S transition of the cell cycle.

Thus, Western blot analysis was performed to assess the protein expression of key regulatory factors associated with the G1 phase. Consistent with our flow cytometry data, the results showed that PSMC2 downregulation caused the accumulation of p21 and markedly decreased the expression of cyclin D1 (Figure 5C). Cyclin D1 is a protein required for progression through the G1 phase of the cell cycle (23). These results demonstrate that PSMC2 facilitates cell proliferation via the induction of the G1/S phase transition.

### PSMC2 Regulates the Expression of p21 at the Transcription Level in HCC

**Figure 6:**
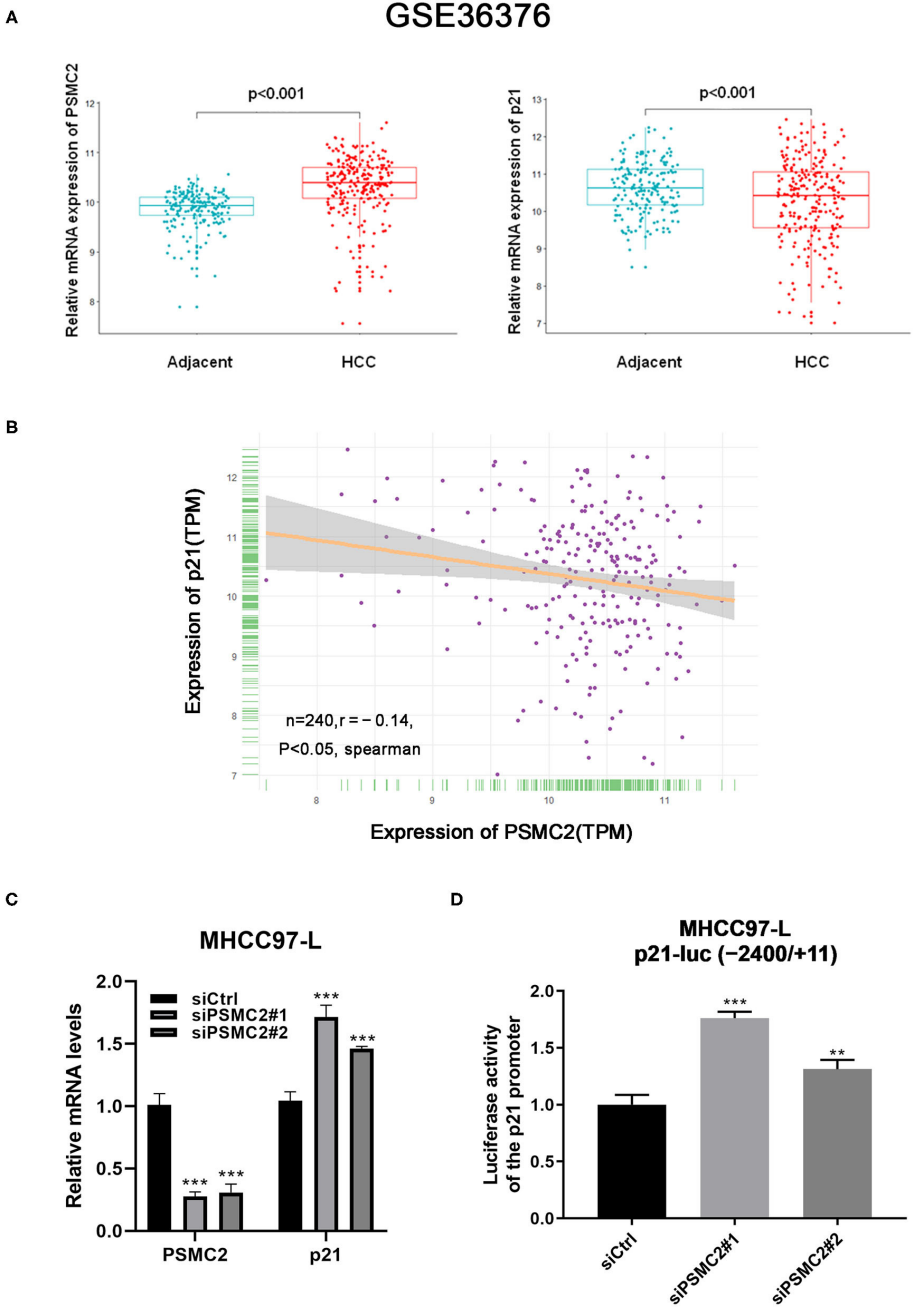
PSMC2 regulates the expression of p21 at the transcription level in HCC. **(A)** PSMC2 mRNA expression was upregulated in cancerous tissue compared to adjacent noncancerous tissue (*p* < 0.001, Student's t test), p21 were downregulated in cancerous tissue (*p* < 0.001). **(B)** Expression of PSMC2 was negatively correlated with p21 in 240 HCC samples. **(C)** Downregulation of PSMC2 increased the mRNA expression of p21. **(D)** Downregulation of PSMC2 enhanced the promoter activity of p21. All experiments were carried out three times independently. Data are mean ± standard deviation. **P < 0.01, ***P < 0.001.

P21 plays an important role in cell growth and the cell cycle (24). Considering the previous results, we assessed whether PSMC2 regulates the expression of p21 at the transcriptional level. We performed bioinformatics analysis of publicly available datasets from the Gene Expression Omnibus (GEO) database (specifically, the GSE36376 dataset, which contains data for 240 HCC samples and 193 adjacent non-cancerous tissues). Notably, PSMC2 mRNA expression was upregulated in cancerous tissue compared with adjacent non-cancerous tissue (*P* < 0.001, Student's *t*-test). In contrast, p21 was downregulated in cancerous tissue (*P* < 0.001, Figure 6A). Moreover, the analysis showed that the expression of PSMC2 was negatively correlated with that of p21 in 240 HCC samples (*r* = −0.14, *P* < 0.05, Spearman correlation; Figure 6B). On the other hand, qRT-PCR showed that the downregulation of PSMC2 increased the mRNA expression of p21 (Figure 6C). According to the current results, we speculate that PSMC2 affects the activity of the p21 promoter. Thus, we used the p21-luc (−2,400/+11) plasmid for dual-luciferase reporter assays. The dual-luciferase reporter assay revealed significant luciferase activity in siPSMC2-transfected cells vs. the control cells (Figure 6D). These results showed that the downregulation of PSMC2 increased the activity of the p21 promoter, which is consistent with our prediction that PSMC2 regulates the expression of p21 at the transcriptional level in HCC.

### Downregulation of p21 Reverses the Inhibition of Proliferation and the Cell Cycle Induced by PSMC2 Knockdown

**Figure 7:**
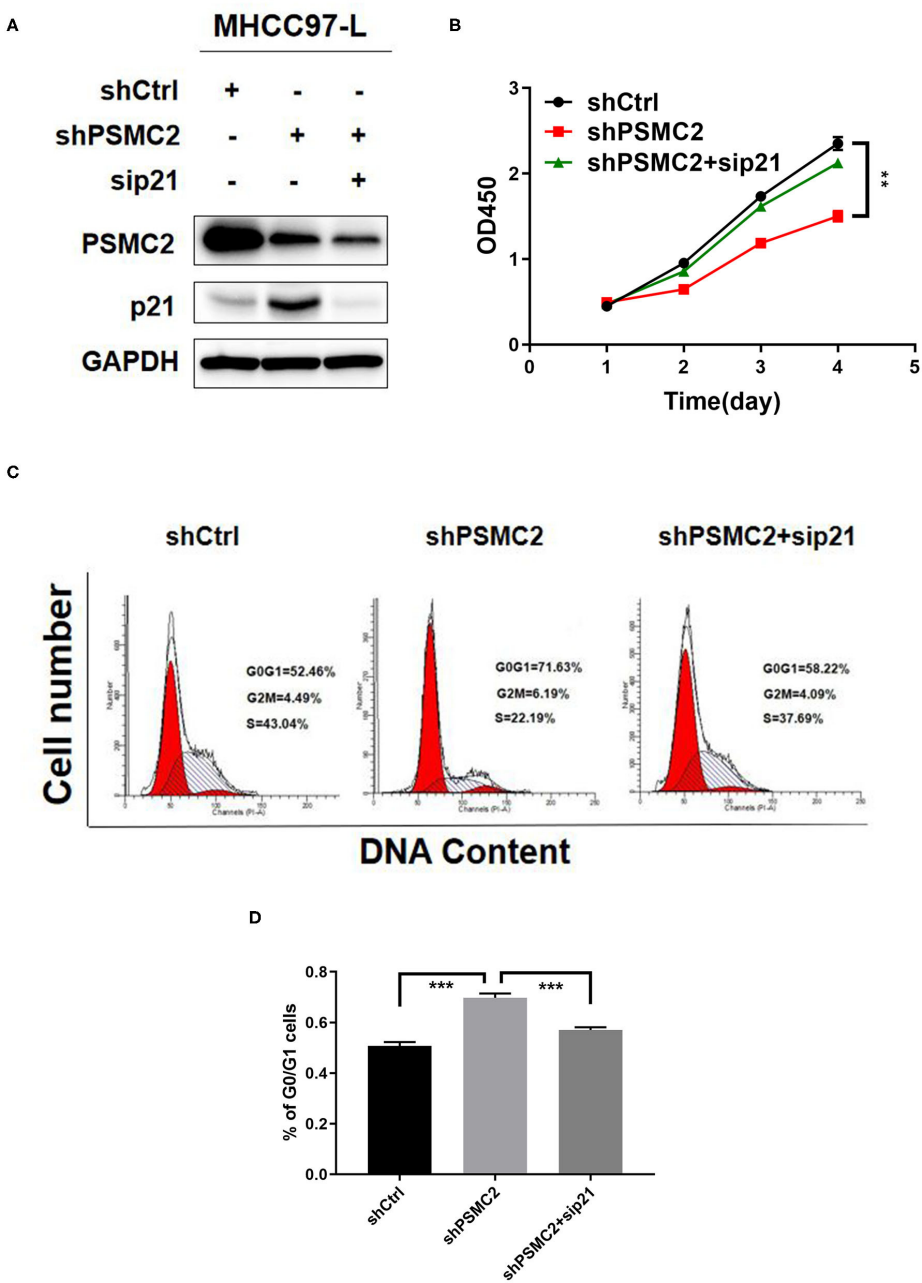
Downregulation of p21 reverses the inhibition of proliferation and cell cycle induced by PSMC2 knockdown. **(A)** PSMC2 and p21 knockdown in MHCC97-L stable cells was confirmed at the protein level by western blotting. **(B–D)** The inhibition of proliferation and cell cycle caused by PSMC2 knockdown was regressed by transient transfection of p21 siRNA in MHCC97-Lstable cells. Data are shown as mean ± standard deviations from three independent experiments. **P < 0.01, ***P < 0.001.

To assess the requirement for p21 in the inhibition of proliferation and the cell cycle induced by PSMC2 silencing, we silenced p21 in PSMC2-depleted MHCC97-L cells (Figure 7A). The results showed that the inhibition of proliferation and the cell cycle caused by PSMC2 knockdown was attenuated by transient transfection of p21 siRNA in MHCC97-L cells (Figures 7B–D). These results demonstrate that p21 plays a crucial role in PSMC2-regulated HCC cell proliferation and cell cycle progression.

### PSMC2 Promotes Tumor HCC Growth *in vivo*

**Figure 8:**
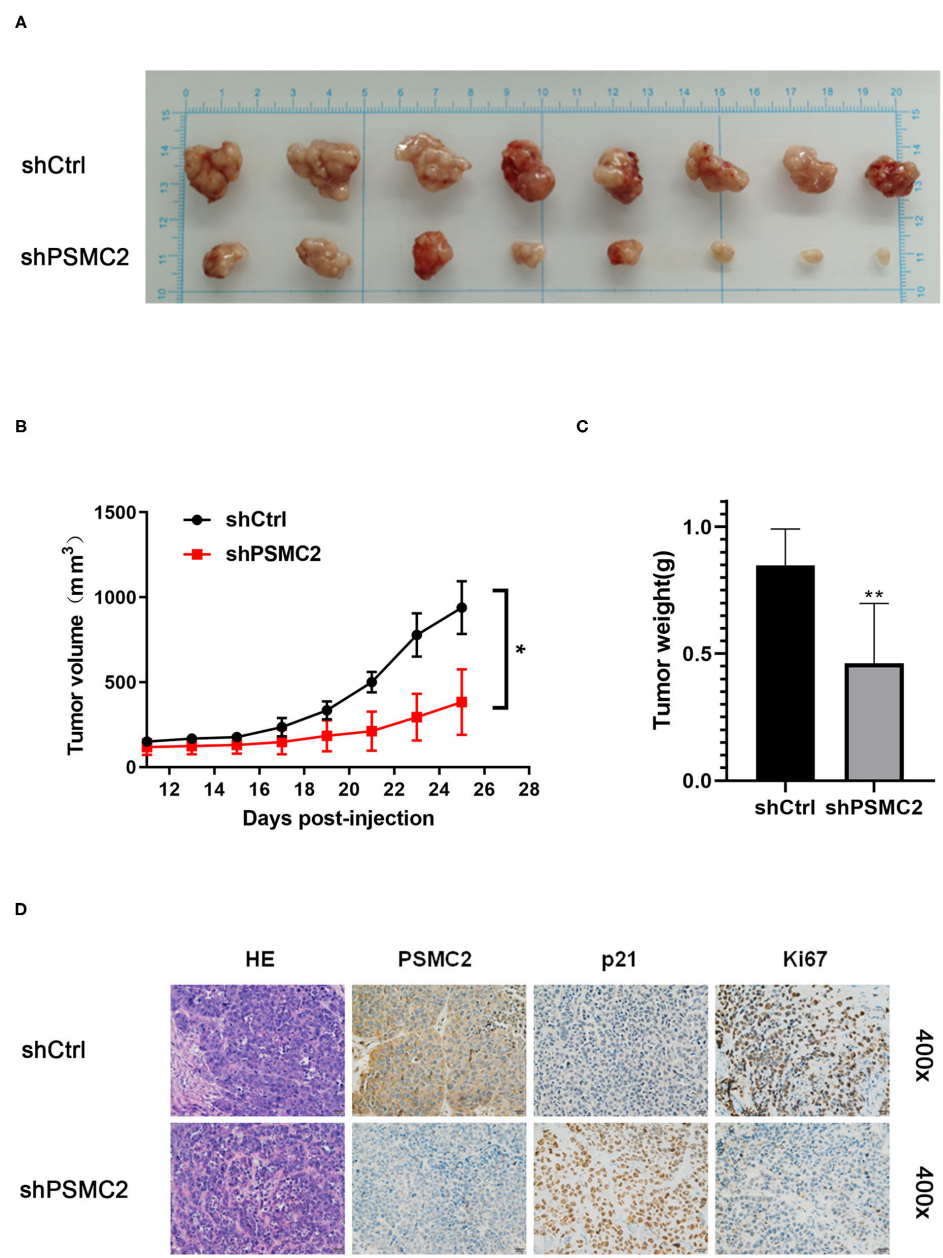
Knockdown of PSMC2 restrains the tumor formation of HCC cells *in vivo*. **(A)** Twenty-five days after injection, the mice were killed and the xenograft tumors were collected. **(B)** Tumor volumes of xenografts in nude mice. Xenografts volumes were calculated with the following formula: V = a × (b × b)/2. **(C)** The weight of the xenograft tumors was analyzed. **(D)** The tumor sections were performed immunochemistry staining by antibody against PSMC2, p21, and Ki67; representative images were shown (400 × magnification). Data are mean ± standard deviation. *P < 0.05, **P < 0.01.

To confirm the effects of PSMC2 on tumor growth *in vivo*, we generated a xenograft model by injecting nude mice with the same number of shPSMC2 or shCtrl MHCC97-L cells to assess the effects of PSMC2 on HCC cell growth (Figure 8A). Consistent with the *in vitro* assays, mice xenografted with shPSMC2 cells formed smaller tumors (in terms of size and weight) *in vivo* than the control mice (Figures 8B,C). Furthermore, IHC staining of tissue sections from tumors derived from shPSMC2 and shCtrl-transfected cells was performed to evaluate the expression of PSMC2, p21, and the nuclear cell proliferation marker Ki-67. The representative images showed that compared with the control conditions, the downregulation of PSMC2 resulted in weaker PSMC2 and Ki-67 staining intensities in the excised tumors but a stronger p21 staining intensity (Figure 8D).

In conclusion, these results clearly demonstrate that high PSMC2 expression enhances tumor growth *in vitro* and *in vivo*.

## Discussion

PSMC2, a key member of the 19S RP, a component of the 26S proteasome complex, has been studied in various cancers. The 26S proteasome complex regulates a multitude of cellular processes, such as cell cycle progression, apoptosis, signal transduction, DNA repair and gene transcription, which are linked to the progression of cancer (25). As these cellular proteins can have direct or indirect regulatory impacts on cancer development, the proteasome has become a potential target for the treatment of multiple cancers. Currently, an increasing number of proteasome inhibitors are being selected to treat malignant diseases in clinical trials (26, 27). Based on the intimate relationship between PSMC2 and the 26S proteasome complex, we conducted elaborate research to explore the correlation of PSMC2 with HCC. First, we observed that PSMC2 is usually highly expressed in HCC tissues compared with paired adjacent non-cancerous tissues. Furthermore, our data indicated that elevated expression of PSMC2 is significantly associated with a larger tumor size and with poor OS and DFS. In addition, univariate and multivariate Cox regression analyses showed that PSMC2 is an independent prognostic biomarker for HCC. These findings suggest that PSMC2 plays a crucial role in HCC progression and could also be a potential therapeutic target.

To further understand the biological function of PSMC2 in HCC aggressiveness, we investigated the malignant features of PSMC2 in HCC cell lines. The TMA analysis showed that PSMC2 is closely related to tumor size, and this effect may be due to the influence of PSMC2 on cell proliferation, apoptosis, and necrosis. Decreased apoptosis and increased cell proliferation are hallmarks of cancer that have been observed over the last decades. Therefore, we carried out related cytology experiments. Our current study showed that the downregulation of PSMC2 in HCC cells can promote apoptosis and inhibit proliferation. Cell cycle dysregulation may lead to enhanced cell proliferation (28). Subsequently, we found that the downregulation of PSMC2 in HCC cells led to cell cycle arrest at the G0/G1 phase. To our knowledge, the cyclin-dependent kinase inhibitor p21 is a cell cycle inhibitor regulated in P53-dependent or P53-independent ways (29–31). Previous studies have shown that under various stress stimuli, p21 mainly functions by blocking G1 or G2 progression and therefore is a crucial regulator of cancer cell growth inhibition (24, 32). Cyclin D1, a key regulator in the G1-to-S-phase transition, is overexpressed in a large fraction of human cancers (33, 34). Previous studies have demonstrated that cyclin D1 binds and sequesters p21, thereby allowing the progression from G1 to S phase (35, 36). Therefore, consistent with our expectation, Western blotting showed that downregulation of PSMC2 dramatically caused the accumulation of p21 and the depletion of cyclin D1 at the protein level.

Following the above studies, we determined whether PSMC2 regulates the expression of p21 at the transcriptional level. qRT-PCR showed that the downregulation of PSMC2 increased the mRNA expression of p21. On the other hand, the GEO database (GSE36376) showed a significant negative correlation between PSMC2 and p21 mRNA expression. Moreover, our dual-luciferase reporter assay revealed that downregulation of PSMC2 increased the activity of the p21 promoter, suggesting that the pivotal role of PSMC2 in regulating the expression of p21 was exerted at the transcription level in HCC. The p53/p21 complex is a functional unit that acts on multiple cell components, leading to cell cycle arrest (37–39). P53 is a tumor suppressor gene that functionally acts as the primary “guardian of the genome.” It has multiple functions, including cell cycle control, apoptosis and maintenance of genomic stability (40, 41). The ubiquitin proteasome pathway is critical in restraining the activities of the p53 tumor suppressor (42). Thus, we had previously explored whether PSMC2 mediated the ubiquitination of P53 to affect the downstream key gene p21. However, Western blotting analysis showed that PSMC2 did not enhance the ubiquitination and stability of P53 (data not shown). There are several scenarios in which the p21 expression pattern is independent of p53; some factors involved in these scenarios include Sp1/Sp3, Smads, Ap2, signal transducers and activators of transcription (STATs), BRCA1, E2F-1/E2F-3, and CAAT/enhancer binding protein α and β (43–46). Consequently, PSMC2 may inhibit the expression of P21 via a pathway that does not rely on P53-mediated regulation. We will conduct further research to obtain more direct evidence.

In conclusion, our data suggest that PSMC2 promotes HCC cell proliferation and cycle progression through the p21/cyclin D1 signaling pathway and provide novel evidence supporting the clinical and biological significance of PSMC2 in HCC. Therefore, PSMC2 could be a potential diagnostic and therapeutic target in HCC.

## Data Availability Statement

Publicly available datasets were analyzed in this study. This data can be found here: https://www.ncbi.nlm.nih.gov/geo/query/acc.cgi?acc=GSE36376.

## Ethics Statement

The studies involving human participants were reviewed and approved by The ethics committee of Nanjing Medical University. The patients/participants provided their written informed consent to participate in this study. The animal study was reviewed and approved by the Institutional Committee on Animal Care of Nanjing Medical University.

## Author Contributions

YL and XWu: study concept and design. YL and HC: drafting of the manuscript and statistical analysis. JB and XWu: critical revision of the manuscript for important intellectual content. XWu: obtained funding. XL, FZ, and LK: administrative, technical, or material support. XWa: study supervision. All authors have read and approved the final version of the manuscript.

## Conflict of Interest

The authors declare that the research was conducted in the absence of any commercial or financial relationships that could be construed as a potential conflict of interest.

## Abbreviations

PSMC2: proteasome 26S subunit ATPase 2
HCC: hepatocellular carcinoma
TMA: tissue microarray
CCK-8: cell counting kit-8
AFP: alpha fetoprotein
IHC: Immunohistochemistry
SiRNA: Small interfering RNA
QRT-PCR: quantitative real-time PCR
GEO: Gene Expression Omnibus.
